# Association of Type of Treatment Facility With Overall Survival After a Diagnosis of Head and Neck Cancer

**DOI:** 10.1001/jamanetworkopen.2019.19697

**Published:** 2020-01-24

**Authors:** Ryan M. Carey, Ramie Fathy, Ravi R. Shah, Karthik Rajasekaran, Steven B. Cannady, Jason G. Newman, Said A. Ibrahim, Jason A. Brant

**Affiliations:** 1 Department of Otorhinolaryngology–Head and Neck Surgery, Perelman School of Medicine, University of Pennsylvania, Philadelphia; 2 currently a medical student at Perelman School of Medicine, University of Pennsylvania, Philadelphia; 3 Department of Healthcare Policy and Research, Weill Cornell Medicine, New York, New York

## Abstract

**Importance:**

Patients with head and neck cancer receive care at academic comprehensive cancer programs (ACCPs), integrated network cancer programs (INCPs), comprehensive community cancer programs (CCCPs), and community cancer programs (CCPs). The type of treatment facility may be associated with overall survival.

**Objective:**

To examine whether type of treatment facility is associated with overall survival after a diagnosis of head and neck cancer.

**Design, Setting, and Participants:**

This population-based retrospective cohort study included patients from the National Cancer Database, a prospectively maintained, hospital-based cancer registry of patients treated at more than 1500 US hospitals. Participants were diagnosed with malignant tumors of the head and neck from January 1, 2004, through December 31, 2016. Data were analyzed from May 1 through November 30, 2019.

**Exposures:**

Treatment at facilities classified as ACCPs, INCPs, CCCPs, or CCPs.

**Main Outcomes and Measures:**

Overall survival after diagnosis and treatment of head and neck cancer was the primary outcome. The secondary outcome was the odds of receiving treatment at ACCPs and INCPs vs CCCPs and CCPs. Multivariable Cox proportional hazards regression and univariable and multivariable logistic regression models were used for analysis.

**Results:**

A total of 525 740 patients (368 821 men [70.2%]; mean [SD] age, 63.3 [14.0] years) were diagnosed with malignant tumors of the head and neck during the study period. Among them, 36 595 patients (7.0%) were treated at CCPs; 174 658 (33.2%), at CCCPs; 232 867 (44.3%), at ACCPs; and 57 857 (11.0%), at INCPs. The median survival for patients with aerodigestive cancers was 69.2 (95% CI, 68.6-69.8) months; salivary gland cancers, 107.2 (95% CI, 103.9-110.2) months; and skin cancers, 113.2 (95% CI, 111.4-114.6) months. Improved overall survival was associated with treatment at ACCPs (hazard ratio [HR], 0.89; 95% CI, 0.88-0.91), INCPs (HR, 0.94; 95% CI, 0.92-0.96), and CCCPs (HR, 0.94; 95% CI, 0.92-0.95) compared with CCPs. Compared with patients with private insurance, those with government insurance (odds ratio [OR], 1.35; 95% CI, 1.29-1.41), no insurance (OR, 1.12; 95% CI, 1.09-1.16), or Medicaid (OR, 1.17; 95% CI, 1.14-1.20) were more likely to receive treatment at ACCPs and INCPs, whereas patients with Medicare were less likely to receive treatment at ACCPs and INCPs (OR, 0.95; 95% CI, 0.94-0.97). Compared with white patients, black (OR, 1.55; 95% CI, 1.52-1.59) and Asian (OR, 1.56; 95% CI, 1.49-1.63) patients were more likely to receive care at ACCPs and INCPs. Compared with patients from lower-income areas, patients from high-income areas were more likely to receive treatment at ACCPs and INCPs (OR, 1.25; 95% CI, 1.22-1.28).

**Conclusions and Relevance:**

These findings suggest that treatment at ACCPs and INCPs was associated with a better overall survival rate in patients with head and neck cancer. Key social determinants of health such as race/ethnicity, socioeconomic status, and type of insurance were associated with receiving treatment at ACCPs and INCPs.

## Key Points

**Question:**

Is the type of treatment facility associated with overall survival in patients with head and neck cancer?

**Findings:**

In this cohort study of 525 740 patients diagnosed with malignant neoplasms of the head and neck in the National Cancer Database from 2004 to 2016, treatment at academic comprehensive cancer programs, integrated network cancer programs, and comprehensive community cancer programs was associated with better overall survival rates than treatment at community cancer programs.

**Meaning:**

This study’s findings suggest that improved understanding of socioeconomic differences and oncologic treatment disparities may improve clinical outcomes in head and neck cancer.

## Teaser

This cohort study uses data from the National Cancer Database to examine whether the type of treatment facility is associated with overall survival among patients with a diagnosis of head and neck cancer.

## Introduction

Malignant tumors of the head and neck account for approximately 4% of all cancers in the United States and involve collaboration between multiple specialties for optimal treatment.^1^ Owing to the highly specialized management of head and neck cancer, treatment factors such as hospital volume and teaching status are often thought to contribute to variation in patient outcomes.^2,3,4^ Physician and institutional variables such as technical ability, treatment modalities offered, and multidisciplinary support and patient factors such as ethnicity, socioeconomic status, and insurance status can vary widely between types of institutions, all of which may affect outcomes.^2,3,4,5,6^ Despite these disparities, no consensus exists on the differences between the quality of care delivered at teaching and nonteaching hospitals.^7,8^

A study from Puram and Bhattacharyya^9^ using data from the Nationwide Inpatient Sample analyzed quality metrics for patients undergoing surgery for head and neck cancer from academic and nonacademic institutions. Their study found that academic institutions had a greater proportion of patients with a history of radiotherapy, high-acuity procedures, and greater comorbidity scores. Controlling for these variables and others showed a slightly increased length of stay and wound infection rates at academic hospitals. A separate study from the Nationwide Inpatient Sample by Dimick et al^2^ found that teaching hospitals had lower operative mortality rates for complex surgical procedures (esophageal, hepatic, or pancreatic resections); however, they found no difference in operative mortality after controlling for hospital volume on multivariate analysis. Eskander et al^3^ conducted a meta-analysis comparing outcomes in head and neck cancer at high- and low-volume centers. They demonstrated better overall survival among patients treated by high-volume hospitals and surgeons than among patients treated by low-volume hospitals and surgeons. However, to our knowledge, no studies have broadened the comparison of treatment facility type and outcomes beyond teaching vs nonteaching facilities.

Therefore, the primary objective of this study was to use data from the National Cancer Database (NCDB) to evaluate factors that contribute to overall survival in patients with head and neck cancers by comparing outcomes for academic comprehensive cancer programs (ACCPs), integrated network cancer programs (INCPs), comprehensive community cancer programs (CCCPs), and community cancer programs (CCPs). Academic comprehensive cancer programs were facilities that participated in postgraduate medical education in at least 4 program areas (including general surgery and internal medicine) and had more than 500 newly diagnosed cancer cases each year. Integrated network cancer programs were defined by having a unified cancer committee with coordinated practice locations and health care professionals, and training of resident physicians was optional. Comprehensive CCPs and CCPs were facilities where training of resident physicians was optional and included more than 500 and 100 to 500 newly diagnosed cancer cases each year, respectively. Furthermore, we investigated demographic and socioeconomic factors for their association with the type of facility where treatment was administered.

## Methods

### Study Sample

The NCDB data set is a joint project of the Commission on Cancer of the American College of Surgeons and the American Cancer Society. The data used in this study are derived from a deidentified NCDB file. This study was determined to be exempt by the University of Pennsylvania institutional review board and did not require informed consent for the use of deidentified data. This report follows the Strengthening the Reporting of Observational Studies in Epidemiology (STROBE) reporting guideline.^10^

Data were obtained from the NCDB from January 1, 2004, through December 31, 2016, and analyzed from May 1 through November 30, 2019. The NCDB uses the *International Classification of Diseases for Oncology, Third Edition*,^11^ which is similar but not identical to the *International Statistical Classification of Diseases and Related Health Problems, Tenth Revision*. The NCDB was queried for primary site codes in the head and neck, excluding thyroid (eTable in the Supplement). Site codes were further divided into aerodigestive, salivary gland, and skin. Only cases designated as “malignant neoplasms stated or presumed to be primary” (behavior code of 3) were included, and no histology type was excluded. To avoid confounding of different surgical procedures and ensure that the surgical procedures were on the primary site, cases were excluded for surgery at a distant site. Cases were also excluded if diagnosis and treatment were performed at different facilities or if they were missing data on mortality status at follow-up.

### Primary and Secondary Outcome Variables

The primary outcome of interest was overall survival. Demographic and socioeconomic factors contributing to receiving treatment at ACs and INCPs vs CCCPs and CCPs served as the secondary outcome, calculated as odds ratios (ORs). Descriptive statistics were calculated.

### Statistical Analysis

Factors associated with overall survival were evaluated using multivariable Cox proportional hazards regression models. Variables to include in the models were selected a priori based on consensus of the contributing authors and included age, sex, race/ethnicity, educational level, median income, housing area (ie, urban vs rural), geographic area, insurance type, facility location, facility type, Charlson/Deyo comorbidity score, and year of diagnosis. Definitions of the variables have been described in prior studies.^12,13^ Housing areas were defined based on the size of each facility’s county (metropolitan, >250 000 residents; urban, 2500-250 000 residents; and rural, <2500 residents). The population educational level was determined from the 2012 American Community Survey based on the percentage of adults in the patient’s zip code who did not have a high school diploma. The primary insurance provider at the time of cancer diagnosis was used for determining insurance status.

Additional analyses of overall survival by facility type for subsets of patients receiving surgical therapy or radiotherapy were also performed. To determine associations of demographic and socioeconomic factors with type of facility, multivariable logistic models with the same variables described above were used to compare the combined group of ACCPs and INCPs with the combined group of CCPs and CCCPs. Statistical analyses were performed with R, version 3.4.1 statistical software (R Project for Statistical Computing), via RStudio, version 1.1.23 statistical software (RStudio, Inc). Missing data were removed from survival and logistic models. Two-sided *P* < .05 indicated statistical significance.

## Results

### Baseline Characteristics of the Sample

A total of 581 726 patients met facility and behavior criteria with complete data for follow-up. Of these, 28 470 were excluded for surgery at a distant site, and 27 516 were excluded for not receiving treatment at the facility where they were diagnosed. After exclusions, 525 740 participants (368 821 men [70.2%] and 156 919 women [29.8%]; mean [SD] age, 63.3 [14.0] years) were included in the final analysis. Among these, 389 495 patients (74.1%) had aerodigestive cancers; 36 700 (7.0%), cancers of the salivary gland; and 99 545 (18.9%), skin cancers. Most skin cancers had aggressive histologic subtypes, including approximately 80% melanoma subtypes and 6% Merkel cell carcinoma. The median survival for patients with aerodigestive cancers was 69.2 (95% CI, 68.6-69.8) months; salivary gland cancers, 107.2 (95% CI, 103.9-110.2) months; and skin cancers, 113.2 (95% CI, 111.4-114.6) months. The Table lists the various demographic variables included in the study. During the study period, 36 595 patients (7.0%) were treated at CCPs; 174 658 (33.2%), at CCCPs; 232 867 (44.3%), at ACCPs; and 57 857 (11.0%), at INCPs.

**Table.** Demographic Information for All Participants^a^

Characteristic	Patient Data	Cox Proportional Hazards Regression		Logistic Regression		Survival, Median (95% CI), mo	Proportion Treated at ACCP or INCP, No. (%)
		HR (95% CI)	*P* Value	OR (95% CI)	*P* Value		
All, No. (%)	525 740 (100)	NA	NA	NA	NA	79.3 (78.8-79.9)	304 509 (57.9)
Age, mean (SD), y	63.29 (13.97)	1.03 (1.03-1.03)	<.001	0.99 (0.99-0.99)	<.001	NA	304 509 (57.9)
Year of diagnosis	2009	0.99 (0.99-0.99)	<.001	1.03 (1.03-1.03)	<.001	NA	304 509 (57.9)
Sex, No. (%)							
Male	368 821 (70.2)	1 [Reference]	NA	1 [Reference]	NA	75.4 (74.9-76.0)	212 552 (57.6)
Female	156 919 (29.8)	0.86 (0.86-0.87)	<.001	1.05 (1.04-1.07)	<.001	89.0 (88.0-90.1)	91 955 (58.6)
Race/ethnicity, No. (%)							
White	458 344 (87.2)	1 [Reference]	NA	1 [Reference]	NA	81.8 (81.2-82.3)	258 735 (56.4)
Black	45 641 (8.7)	1.36 (1.34-1.38)	<.001	1.55 (1.52-1.59)	<.001	44.0 (42.8-45.3)	30 771 (67.4)
Other/unknown	10 673 (2.0)	0.94 (0.91-0.97)	<.001	1.88 (1.80-1.97)	<.001	105.4 (97.8-111.1)	7665 (71.8)
Asian	111 082 (2.1)	0.96 (0.93-0.99)	.01	1.56 (1.49-1.63)	<.001	123.2 (116.6-130.3)	75 369 (67.8)
Facility location, No. (%)							
Northeast	102 434 (19.5)	1 [Reference]	NA	1 [Reference]	NA	80.5 (79.4-81.6)	70 239 (68.6)
South	149 793 (28.5)	1.02 (1.01-1.04)	<.001	0.60 (0.59-0.61)	<.001	69.7 (68.7-70.6)	82 761 (55.3)
Midwest	170 450 (32.4)	1.03 (1.01-1.04)	<.001	0.72 (0.71-0.74)	<.001	72.5 (71.8-73.4)	97 941 (57.5)
West	79 300 (15.1)	0.98 (0.97-0.99)	.007	0.47 (0.46-0.47)	<.001	84.4 (83.0-85.8)	39 785 (50.2)
Missing	23 763 (4.5)	NA	NA	NA	NA	NA	NA
Housing area, No. (%)							
Metropolitan	421 668 (80.2)	1 [Reference]	NA	1 [Reference]	NA	81.0 (80.4-81.5)	254 814 (60.4)
Urban	79 907 (15.2)	0.95 (0.94-0.96)	<.001	0.64 (0.63-0.65)	<.001	72.0 (70.9-73.3)	37 141 (46.5)
Rural	10 544 (2.0)	0.91 (0.89-0.94)	<.001	0.53 (0.51-0.55)	<.001	70.2 (67.2-73.8)	4363 (41.4)
Missing	13 621 (2.6)	NA	NA	NA	NA	NA	7985 (58.6)
Educational level, No. (%)^b^							
≥21.0	90 360 (17.2)	1 [Reference]	NA	1 [Reference]	NA	59.2 (58.1-60.2)	51 062 (56.5)
13.0-20.9	138 695 (26.4)	0.98 (0.97-0.99)	.001	0.94 (0.92-0.96)	<.001	68.9 (68.0-69.9)	76 560 (55.2)
7.0-12.9	170 021 (32.3)	0.93 (0.92-0.94)	<.001	0.93 (0.92-0.95)	<.001	83.0 (82.1-83.9)	97 422 (57.3)
<7.0	120 040 (23.6)	0.84 (0.83-0.86)	<.001	1.05 (1.03-1.08)	<.001	104.1 (102.8-105.6)	78 538 (65.4)
Missing	2624 (0.5)	NA	NA	NA	NA	NA	1542 (58.8)
Median income, $, No. (%)							
<38 000	96 570 (18.4)	1 [Reference]	NA	1 [Reference]	NA	54.8 (54.0-55.7)	54 301 (56.2)
38 000-47 999	126 076 (24.0)	0.93 (0.92-0.94)	<.001	0.93 (0.92-0.95)	<.001	70.3 (69.4-71.2)	66 152 (52.5)
48 000-62 999	138 875 (26.4)	0.89 (0.87-0.90)	<.001	1.04 (1.02-1.06)	<.001	81.9 (80.9-83.0)	78 964 (56.9)
≥63 000	161 248 (30.7)	0.82 (0.80-0.83)	<.001	1.25 (1.22-1.28)	<.001	103.8 (102.6-105.2)	103 392 (64.1)
Missing	2971 (0.6)	NA	NA	NA	NA	NA	1742 (58.62)
Primary insurance, No. (%)							
Private	208 367 (39.6)	1 [Reference]	NA	1 [Reference]	NA	152.7 (151.0-154.8)	124 687 (59.8)
Not insured	23 117 (4.4)	2.04 (2.00-2.09)	<.001	1.12 (1.09-1.16)	<.001	64.2 (61.8-67.2)	14 157 (61.2)
Medicaid	41 650 (7.9)	2.35 (2.31-2.38)	<.001	1.17 (1.14-1.20)	<.001	43.5 (42.5-45.1)	26 552 (63.8)
Medicare	232 518 (44.2)	1.36 (1.34-1.37)	<.001	0.95 (0.94-0.97)	<.001	50.7 (50.3-51.1)	127 025 (54.6)
Other government	9782 (1.9)	1.46 (1.42-1.51)	<.001	1.35 (1.29-1.41)	<.001	71.9 (68.0-75.4)	6085 (62.2)
Unknown	10 306 (2.0)	1.45 (1.41-1.50)	<.001	1.28 (1.22-1.34)	<.001	NA	6599 (64.0)
Facility type, No. (%)							
CCP	36 595 (7.0)	1 [Reference]	NA	NA	NA	62.8 (61.0-64.1)	NA
CCCP	174 658 (33.2)	0.94 (0.92-0.95)	<.001	NA	NA	71.2 (70.4-72.0)	NA
ACCP	232 867 (44.3)	0.89 (0.88-0.91)	<.001	NA	NA	80.8 (80.1-81.6)	NA
INCP	57 857 (11.0)	0.94 (0.92-0.96)	<.001	NA	NA	74.0 (72.6-75.5)	NA
Missing	23 763 (4.5)	NA	NA	NA	NA	NA	NA
Charlson/Deyo comorbidity score, No. (%)							
0	415 022 (78.9)	1 [Reference]	NA	1 [Reference]	NA	92.1 (91.4-92.7)	243 327 (58.6)
1	82 579 (15.7)	1.30 (1.29-1.32)	<.001	0.91 (0.90-0.93)	<.001	52.1 (51.2-53.0)	45 906 (55.6)
2	20 128 (3.8)	1.69 (1.66-1.72)	<.001	0.86 (0.84-0.89)	<.001	31.9 (30.9-32.9)	10 779 (53.6)
3	8011 (1.5)	2.09 (2.03-2.15)	<.001	0.96 (0.92-1.01)	.09	23.5 (22.4-24.5)	4611 (57.6)

Abbreviations: ACCP, academic comprehensive cancer program; CCCP, comprehensive community cancer program; CCP, community cancer program; HR, hazard ratio; INCP, integrated network cancer program; NA, not applicable; OR, odds ratio.

^a^ Demographic variables are given with values from multivariable Cox proportional hazards regression models of overall survival and logistic regression for treatment at ACCPs and INCPs compared with CCPs or CCCPs. Also shown are the median survival and proportion treated at ACCPs and INCPs.

^b^ Calculated as the percentage of adults without a high school diploma in the patient’s zip code.

### Results From Multivariable Models

**Figure 1.**
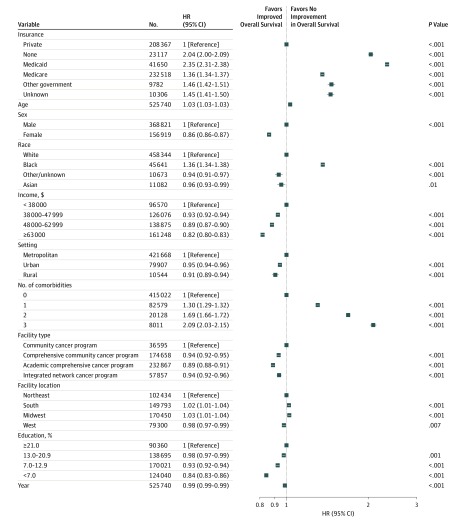
Factors Associated With Overall Survival on Multivariable Analysis Calculated using Cox proportional hazards regression analysis. Education is calculated as the percentage of adults without a high school diploma in the patient’s zip code. Hazard ratios (HRs) less than 1.00 represent improved overall survival.

**Figure 2.**
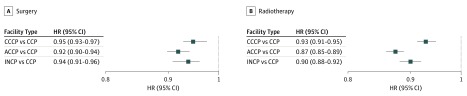
Univariable Log-Rank Analyses by Facility Type for Patients Receiving Surgery and Radiotherapy Hazard ratios (HRs) of less than 1.00 represent improved overall survival. ACCP indicates academic comprehensive cancer program; CCCP, comprehensive community cancer program; CCP, community cancer program; and INCP, integrated network cancer program.

Several variables were significantly associated with overall survival on multivariable analysis. Specifically, treatment at ACCPs (hazard ratio [HR], 0.89; 95% CI, 0.88-0.91), INCPs (HR, 0.94; 95% CI, 0.92-0.96), and CCCPs (HR, 0.94; 95% CI, 0.92-0.95) were associated with improved overall survival on multivariable analysis compared with CCPs (Figure 1 and Table). Results of univariable analyses by facility type for subsets of patients receiving surgical therapy with or without radiotherapy and for patients receiving radiotherapy with or without surgery are shown in Figure 2. Multivariable subanalysis for patients receiving surgical therapy did not demonstrate statistically significant differences in overall survival at different facility types. Multivariable analysis for patients receiving radiotherapy demonstrated improved overall survival at ACCPs (HR, 0.95; 95% CI, 0.93-0.97), INCPs (HR, 0.95; 95% CI, 0.93-0.98), and CCCPs (HR, 0.96; 95% CI, 0.94-0.98).

**Figure 3.**
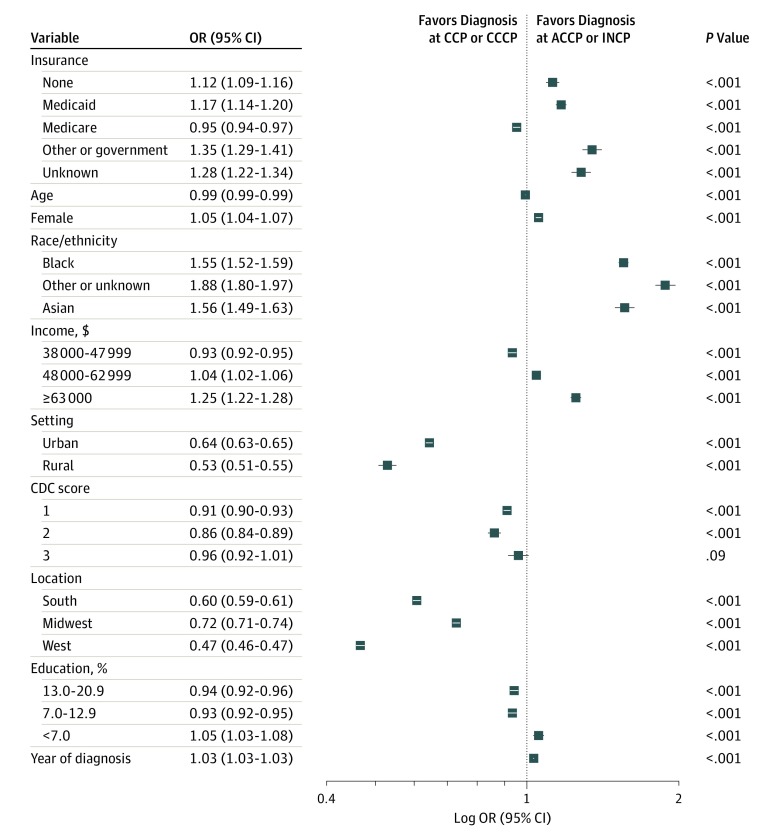
Factors Associated With Receiving Treatment at Academic Comprehensive Cancer Programs (ACCPs) and Integrated Network Cancer Programs (INCPs) Odds ratios (ORs) of less than 1.00 indicate lower odds of being diagnosed with head and neck cancer at ACCPs or INCPs compared with community cancer programs (CCPs) or comprehensive community cancer programs (CCCPs). Education is calculated as the percentage of adults without a high school diploma in the patient’s zip code. CDC score indicates Charlson/Deyo comorbidity score.

Multiple factors were associated with receiving care at ACCPs and INCPs (Figure 3 and Table). Compared with private insurance, having Medicaid (OR, 1.17; 95% CI, 1.14-1.20), no insurance (OR, 1.12; 95% CI, 1.09-1.16), and other government insurance (OR, 1.35; 95% CI, 1.29-1.41) were associated with greater odds of receiving treatment at ACCPs and INCPs, whereas having Medicare was associated with decreased odds (OR, 0.95; 95% CI, 0.94-0.97). Black patients (OR, 1.55; 95% CI, 1.52-1.59) and Asian patients (OR, 1.56; 95% CI, 1.49-1.63) were more likely to receive care at ACCPs and INCPs compared with white patients. Compared with the lowest income bracket, patients with an annual income of $63 000 or greater were more likely to receive treatment at ACCPs and INCPs (OR, 1.25; 95% CI, 1.22-1.28). Patients with the lowest education levels were more likely to receive treatment at ACCPs and INCPs (OR, 1.05; 95% CI, 1.03-1.08). Patients from urban (OR, 0.64; 95% CI, 0.63-0.65) and rural (OR, 0.53; 95% CI, 0.51-0.55) communities were less likely to receive care at ACCPs and INCPs compared with individuals from metropolitan areas.

There was a small but consistent and statistically significant trend toward increased diagnosis and treatment at ACCPs and INCPs compared with CCCPs and CCPs over time that appeared to be contributed to primarily by findings for increased diagnosis and treatment at ACCPs. Further analysis of this trend was beyond the scope of the current report.

## Discussion

In this large national database analysis, we found that patients with head and neck cancers who were diagnosed and treated at ACCPs, INCPs, and CCCPs had better overall survival rates compared with patients who received treatment at CCPs. This difference was also seen in the subset of patients receiving radiotherapy as part of their treatment. Specifically, patients who received radiotherapy at ACCPs, INCPs, and CCCPs had improved overall survival compared with those receiving radiotherapy at CCPs. The second key finding of this study was the association between social determinants of health, such as race/ethnicity, income, educational level, and community type, and where one receives treatment.

Univariate analysis of facility type for the subset of patients receiving surgical therapy demonstrated improved overall survival at ACCPs, INCPs, and CCCPs compared with CCPs; however, no significant survival difference was found on multivariable analysis. A previous NCDB analysis of oral cavity cancer by Rubin et al^14^ found that patients treated at ACCPs were more likely to receive surgical treatment and had improved overall survival compared with patients treated at CCPs and CCCPs. Furthermore, those investigators found that patients treated at ACCPs were more likely to undergo neck dissection even after controlling for tumor stage. Based on the work by D’Cruz et al,^15^ Rubin et al^14^ hypothesized that the improved overall survival seen in the ACCP group may have been at least partially affected by the survival benefits from elective neck dissection compared with therapeutic neck dissection in early-stage oral cavity cancer.

Obtaining negative surgical margins is another important principle of head and neck oncologic surgery because it affects the overall prognosis in many cancers.^16,17,18^ The NCDB analysis on oral cavity cancer by Luryi et al^19^ found that positive surgical margins were associated with treatment at nonacademic cancer centers and institutions with lower oral cancer case volume. The present study did not investigate associations between margin status and treatment facility, although these factors may have affected survival.

Treatment of head and neck cancer often requires multiple specialists and significant resources, including facilities capable of delivering radiotherapy. One retrospective study of patients with head and neck cancer diagnosed at a single academic center^6^ found that patients who received their radiotherapy at nonacademic centers were more likely to have earlier-stage cancer and to receive radiotherapy alone instead of chemoradiotherapy. However, there was no difference in recurrence rates or overall survival between the academic and nonacademic treatment groups.^6^

Another study^4^ evaluated 388 patients with mucosal head and neck cancer treated with primary and adjuvant radiotherapy at academic or community centers and found that patients treated at ACCPs had more advanced disease, decreased rates of smoking, a higher median income, and a higher percentage of oropharyngeal tumors. Of note, the 5-year survival rates were higher in patients treated at ACCPs compared with community centers (53.2% vs 32.8%; *P* < .001).^4^ These investigators found no differences in the rate of treatment completion between academic and community centers.

Many studies have compared teaching with nonteaching hospitals,^2,3,7,8,9^ but few have specifically compared ACCPs, INCPs, CCCPs, and CCPs. This distinction is important but difficult to fully interpret because there may be confounding between resident teaching status and hospital case volume. Because resident training at INCPs, CCCPs, and CCPs was optional, it is unclear what proportion of facilities participated in resident training. Both CCCPs and ACCPs were higher-volume facilities compared with CCPs, whereas the number of newly diagnosed cancer cases at INCPs was not explicitly defined in the NCDB.

During the present study, 44.3% of patients were treated at ACCPs and 11.0% at INCPs compared with 33.2% treated at CCPS and 7.0% at CCPs. This study’s findings indicate that black and Asian patients, patients from metropolitan areas, and patients in the lowest quartile of educational level were more likely to be diagnosed at ACCPs and INCPs. These findings may reflect a proximity bias and could be related to the demographic characteristics of individuals who most often live in areas where ACCPs and INCPs tend to be located.

Our multivariable analysis showed worse overall survival for patients with Medicaid, Medicare, no insurance, and other government insurance compared with private insurance. These findings are supported by a study of head and neck cancer by Inverso et al,^5^ who showed that uninsured patients were more likely to present with metastatic disease and had a higher risk of head and neck cancer–specific mortality. We found that, compared with having private insurance, having Medicaid, no insurance, or other government insurance were associated with greater odds of receiving treatment at ACCPs and INCPs, whereas having Medicare was associated with decreased odds. These findings may again reflect a proximity bias of certain facilities or may be owing to a greater willingness of ACCPs and INCPs to treat patients regardless of their insurance status.

Patients in the highest income bracket were more likely to be diagnosed and treated at ACCPs and INCPs. Patients with higher incomes may have had greater means to seek out care at larger tertiary research centers; however, the present analysis could not make that determination. A higher Charlson/Deyo comorbidity score has been shown to be a strong risk factor for poor overall survival in head and neck cancer,^20^ which was again demonstrated in this study.

These findings suggest improved outcomes for patients with head and neck cancer who receive their treatment at teaching institutions and/or higher-volume facilities. However, socioeconomic and health disparities affect where patients ultimately receive their treatment. Improved access to care for patients from lower socioeconomic status may ultimately help improve these individuals’ outcomes.

### Limitations

Our results must be interpreted within consideration of several limitations. First, the NCDB has potential issues with accuracy and confounding. The data are gathered from multiple centers, each with their own standards for data collection and reporting. Confounding is a known issue with data sets such as the NCDB owing to missing clinically relevant variables that cannot be included in analyses.^21^ For example, the NCDB does not include information on tobacco smoking, which is more common in lower socioeconomic classes^22^ and has an important association with head and neck cancer.^23^ Moreover, our multivariable analyses only controlled for the variables that we incorporated into the statistical models. We attempted to control for advances in the treatment of head and neck cancer during the study period by including the year of diagnosis in the multivariable models. However, we were not able to account for differences in treatment techniques such as transoral robotic surgery or intensity-modulated radiotherapy. Also, the primary outcome of interest, overall survival, is prone to confounding due to various patient and disease factors.

## Conclusions

This study’s findings suggest that social factors such as race/ethnicity, income, educational level, and community type were associated with where patients received treatment. Where patients received treatment was associated with their outcomes because patients with head and neck cancers who received treatment at ACCPs, INCPs, and CCCPs had better overall survival compared with those who received treatment at CCPs. Future studies are necessary to improve our understanding of these socioeconomic differences, reduce the disparities that exist in oncologic treatment, and improve overall outcomes.

## Notes

Supplement. **eTable.** *ICD-O-3* Site Codes for Tumors of the Head and Neck
