# Supplementary material for: Association of Type of Treatment Facility With Overall Survival After a Diagnosis of Head and Neck Cancer
Source: JAMA Netw Open. 2020 Jan 24;3(1):e1919697. doi: 10.1001/jamanetworkopen.2019.19697 (PMC6991286; doi:10.1001/jamanetworkopen.2019.19697)
Supplement: Supplement. — eTable. ICD-O-3 Site Codes for Tumors of the Head and Neck [file jamanetwopen-3-e1919697-s001.pdf]

Supplementary Online Content

Carey RM, Fathy R, Shah RR, et al. Association of type of treatment facility with overall survival after a diagnosis of head and neck cancer. *JAMA Netw Open.* 2020;3(1):e1919697. doi:10.1001/jamanetworkopen.2019.19697

eTable. *ICD-O-3* Site Codes for Tumors of the Head and Neck

This supplementary material has been provided by the authors to give readers additional information about their work.

© 2020 Carey RM et al. *JAMA Network Open.*

**eTable.** *ICD-O-3* Site Codes for Tumors of the Head and Neck	
Aerodigestive	C000, C001, C002, C003, C004, C005, C006, C008, C009, C019, C020, C021, C022, C023, C024, C028, C029, C030, C031, C039, C040, C041, C048, C049, C050, C051, C052, C058, C059, C060, C061, C062, C068, C069, C090, C091, C092, C093, C094, C095, C096, C097, C098, C099, C100, C101, C102, C103, C104, C108, C109, C110, C111, C112, C113, C118, C119, C129, C130, C131, C132, C138, C139, C140, C142, C148, C300, C301, C310, C311, C312, C313, C318, C319, C320, C321, C322, C323, C324, C325, C326, C327, C328, C329
Salivary gland	C079, C080, C081, C082, C083, C084, C085, C086, C087, C088, C089
Skin	C440, C441, C442, C443, C444

© 2020 Carey RM et al. *JAMA Network Open.*
